# Hemorrhagic corpus luteum: Clinical management update

**DOI:** 10.4274/tjod.galenos.2020.40359

**Published:** 2020-12-10

**Authors:** Mykhailo V Medvediev, Antonio Malvasi, Sarah Gustapane, Andrea Tinelli

**Affiliations:** 1 Dnipropetrovsk Medical Academy of Health Ministry of Ukraine, Dnipropetrovsk, Ukrain; 2 Santa Maria Hospital, Gvm Care and Research, Clinic of Obstetrics and Gynaecology, Bari, Italy; 3 Veris Delli Ponti Hospital, Clinic of Obstetrics and Gynecology, Scorrano, Lecce, Italy; 4 Veris Delli Ponti Hospital, Chief of Clinic of Obstetrics and Gynecology, Scorrano, Lecce, Italy

**Keywords:** Corpus luteum, ovarian cyst, ectopic pregnancy, laparoscopy

## Abstract

Hemorrhagic corpus luteum (HCL) is an ovarian cyst formed after ovulation and caused by spontaneous bleeding into a corpus luteum (CL) cyst. When HCL rupture happens, a hemoperitoneum results. Clinical symptoms are mainly due to peritoneal irritation by the blood effusion. The differential diagnosis is extensive and standard management is not defined. The authors elaborated a comparison of the differential diagnosis and therapeutic modalities from the laparoscopic approach to nonsurgical, medical options because hemorrhage from HCL is often self-limiting. The authors reviewed all data implicated with the development of HCL, trying to give homogeneity to literature data. The authors analyzed extensive literature data and subdivided the medical approach into many topics. The wait-and-see attitude avoids unnecessary laparoscopic surgery using supportive therapies (antifibrinolytic, analgesics, liquid infusion, transfusions and antibiotic prophylaxis). Surgical therapy: operative management should be laparoscopic, with surgical options such as luteumectomy, ovarian wedge-shaped excision or oophorectomy. Prevention: the possibility to preserve fertility is essential, mainly in patients with bleeding disorders or undergoing anticoagulant therapy; therefore, they need estro-progestinics or GnRH analogues to prevent ovulation and avoid further episodes of HCL. This review will aid physicians in making an early diagnosis of HCL, to avoid unnecessary surgery, and use the most effective treatment.

## Introduction

Ovulation is a physiologic monthly event and may be rarely complicated by rupture of the corpus luteum (CL), which occurs at all stages of a woman’s reproductive life. The CL is formed during the luteal phase of the ovarian cycle. Spontaneous but self-limiting bleeding fills the central cavity, and when bleeding is excessive, the CL enlarges and forms a hemorrhagic CL cyst, which may rupture. Bleeding from a ruptured CL can vary from mild hemorrhage to massive hemoperitoneum, leading the patient to shock and subsequent emergency surgery. Hemorrhagic corpus luteum (HCL) rupture and bleeding are often triggered by exercise, coitus, trauma or a pelvic examination. Clinical symptoms are mainly due to peritoneal irritation by the blood effusion. The differential diagnosis is extensive and includes ectopic pregnancy, adnexal torsion, neoplasm, and pelvic inflammatory disease (PID)^(1,2)^. The actual incidence of HCL is unknown because it is often asymptomatic and escapes the attention of physicians. Hallatt et al.^(1)^ reported a higher prevalence in young women. Most ruptures occur during the secretory phase of the cycle. HCL is one of the differential diagnoses of acute abdomen in women of reproductive age. Although it can occur at any time of life, it is likely to develop in the early period after menarche. A significant correlation between coitus and rupture of the CL was described by Aggarwal et al.^(3)^, showing a traumatic etiology in 16 of 26 patients (61%). An increased frequency of HCL during pregnancy was also observed, with increased risk of spontaneous miscarriage^(1)^.

The authors reviewed the clinical findings of HCL and elaborated a comparison of differential diagnosis and therapeutic modalities from the laparoscopic approach to nonsurgical, medical options.

In this investigation, we reviewed all data implicated with HCL development, such as age, race, heritage, reproductive factors, sex hormone, obesity, diet, smoking, physical activity, stress, talc use, and environmental and other factors. To perform the clinical research, the authors consulted the following scientific databases: PubMed (1966-2019), the Cochrane Library, Google Scholar (1966-2019), EMBASE (1974-2019), Science citation index (1974-2019), The China Journal Full Text Database (1994-2019), Chinese Scientific Journals Full Text Database (1989-2019), Chinese Biomedical Literature Database (1978-2019), and the WANFANG database (1980-2019).

The following key terms were used to access the records: *corpus luteum, corpus luteum rupture, bleeding, cyst, pregnancy, hemorrhagic corpus luteum, corpus luteum rupture*. The authors reviewed all articles relating to the CL and problems related to the CL, such as the onset and complications, the bleeding caused by the CL, and the pharmacologic and surgical treatment. Randomized controlled studies were used when available; otherwise, literature that was the most relevant to the topic was used at the authors’ discretion. Peer-reviewed articles regarding the CL and hemorrhage, sorted by relevance, were included for the aims of this work. Additional articles were identified from the references of retrieved papers. The aim of this extensive review was to provide information about the clinical and surgical data implicated with the development of HCL and its treatment modalities, trying to give homogeneity to literature data.

### Pathophysiology and Risk Factors

According to Novak and Woodruff, bleeding does not normally occur from the stigma because it fills with fibrin. Ovulation is followed by a stage of proliferation or hyperemia, consisting of follicular collapse and luteinization of the granulosa layer, which is devoid of blood vessels. The lumen of the CL still contains no blood. The stage of vascularization then occurs; the granulosa layer is penetrated by blood vessels that fill the cavity of CL with blood^(4)^. If the CL hematoma ruptures, intraperitoneal hemorrhage may occur, especially if a woman’s clotting mechanisms are depressed by anticoagulant therapy or congenital bleeding disorders. In this study, we focused exactly on this type of HCL hemorrhagic cysts.

### Dextro-preponderance

Many studies observed a higher prevalence of HCL on the right ovary.

Aggarwal et al.^(3)^ reported a dextro-preponderance of HCL in 20 of 26 patients (76.9%).

The study of Tang et al.^(5)^ reported a right prevalence of 81.25%, and proposed that the right preponderance was the result of a different venous architecture causing higher venous pressure in the right ovary. Ho et al.^(6)^ observed a rupture of CL in the right ovary in 60 of 91 patients (65.9%).

According to some authors, the presence of the rectosigmoid colon protects the left ovary from trauma, especially during sexual intercourses^(1,3,6)^.

Pain in the right iliac fossa may mimic acute appendicitis, and the same symptoms related to the left quadrant might be underestimated in many patients, and this may contribute to the increased right prevalence of HCL^(6)^.

Fukuda et al.^(7)^ reported a greater ovulatory activity by the right ovary (~ 55%) than the left ovary during its entire reproductive age.

### Bleeding Disorders and Anticoagulant Therapy

Patients with bleeding disorders have a greater risk of extensive hemoperitoneum than patients with normal coagulation function, and the former often require surgery to stop the bleeding.

Many cases of hemoperitoneum have been reported in patients with von Willebrand disease type 1, 2A, 3^(8,9,10,11,12,13,14)^, patients with afibrinogenemia^(15,16,17)^, patients with Glanzmann’s thrombasthenia, patients with hemophilia A^(18)^, hemophilia B, deficiency of factor X and factor XIII, and in patients receiving anticoagulant therapy for antiphospholipid antibody syndrome^(19,20,21,22)^.

Hemorrhage from a ruptured CL cyst should be considered in any woman of reproductive age who is undergoing anticoagulant therapy because it is a potentially life-threatening complication. When ovarian enlargement and hemorrhagic effusion are detected, anticoagulant therapy should be stopped.

In women with known bleeding disorders or receiving anticoagulant therapy, HCL rupture should always be suspected in the presence of lower quadrant abdominal pain^(2,23)^.

Complete coagulation screening is essential for the early identification of patients with bleeding disorders; anamnesis, anticoagulant therapy, and family history can provide important information.

These patients show a higher risk of recurrent HCL and ovulation should be suppressed with either a low-dose oral contraceptive pill (OC), progesterone-only agents or gonadotropin-releasing hormone analogs.

### Clinical Aspects

Rupture of CL may be asymptomatic or associated with the sudden onset of lower abdominal pain. The pain often begins during strenuous physical activity, such as exercise or sexual intercourse, often lasting less than 24 hours.

Symptoms start in a third of patients with intermittent cramps preceding the acute pain, due to hemoperitoneum resulting from the rupture^(1)^.

The cramps are caused by the luteal cavity distension due to the intracystic bleeding and the pain can range from a diffuse tenderness to acute abdomen when the rupture and the consequent hemoperitoneum occurs; even a small peritoneal effusion is large enough to cause real acute abdomen. Other symptoms may include nausea and vomiting caused by visceral reaction due to peritoneal irritation, vaginal bleeding, weakness, hypotension, syncope, and cardiovascular collapse. Visceral pain can also be related to emotional signs such as marked anxiety and autonomic signs such as pallor, sweating, nausea, vomiting, bradycardia or tachycardia. These signs further amplify the symptoms caused by bleeding.

Barel et al.^(24)^ reported abdominal pain as a prevalent and constant symptom in all patients; 10.7% also had fever, 13% had nausea and vomiting, and 4% showed urinary disorders.

It is worth mentioning that it is not always clinically possible to differentiate hemorrhagic cyst and ruptured hemorrhagic cyst. In many cases of RCL, patients remain hemodynamically stable, and a moderate amount of free fluid in the abdominal cavity could be a normal finding in the postovulatory period. For this reason, we discuss both conditions naming it “HCL cyst” in the article.

### Diagnosis

A physical examination of the abdomen and vagina is critical in the first evaluation of the patient. Accurate diagnosis depends on the clinical presentation, the results of tests, and the index of suspicion. A negative pregnancy test is important to exclude ruptured ectopic pregnancy.

Some diagnostic tests are necessary. Primarily, diagnosis requires an ultrasound (US) examination (very useful to inspect the CL and the abdominal effusion), a pregnancy test, a complete blood count, blood clotting tests, and an evaluation of inflammatory markers.

### Laboratory Tests

For a patient’s hemostasis evaluation, the following parameters should be evaluated:

• Prothrombin time (PT): This is used to assess the extrinsic pathway of coagulation. PT is longer in cases of factor VII, X, V, II, and fibrinogen deficiency, and it is essential to evaluate the hepatic synthesis of coagulation factors and vitamin K status, as well as for monitoring anticoagulant therapy.

• Activated partial thromboplastin time (aPTT): The aPTT is used to evaluate factors of the intrinsic and common pathway of coagulation and to monitor therapy with unfractionated heparin; it could be longer in cases of a shortage of one of the intrinsic pathway factors, or in the presence of antiphospholipid syndrome.

• Fibrinogen: This is reduced in case of liver diseases, CID, and massive transfusions, and is increased during inflammation.

• White blood cell (WBC): Barel et al.^(24)^ observed that elevated WBC (11,000 per mL) was related to the severity of the clinical presentation and its value regresses with resolution^(25)^.

• Hemoglobin (Hgb) and hematocrit (Hct): These tend to decrease progressively and proportionally to the amount of peritoneal effusion. Monitoring the changes Hgb levels is essential to assess the development of blood loss to evaluate the possibility for emergency surgery.

• Platelets: generally indicated to investigate the presence of possible thrombocytopenia or thrombocytopathy in patients with an HCL, without a history of bleeding disorders or anticoagulant therapy^(23)^.

The human chorionic gonadotropin free beta-subunit (beta-hCG) is detectable in blood through radioimmunoassay after four weeks of amenorrhea, and in the urine from the sixth and seventh weeks of gestation age. Beta-hCG is essential to identify pregnant patients and in making a differential diagnosis with extrauterine pregnancy or intrauterine gestation.

### Imaging-Ultrasound

US technology and in particular the use of transvaginal imaging has a key-role in the HCL diagnosis^(26,27)^.

The sonographic appearance of a hemorrhagic ovarian cyst can be different in size, thickness of the cyst wall, and internal echo pattern depending on the formation and lysis of the clot^(28)^.

**Figure 1:**
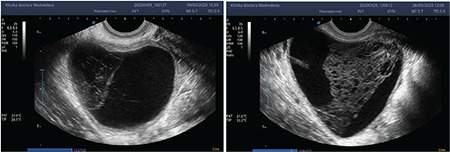
Typical sonographic features of a corpus luteum cyst with hemorrhagic content

Usually, HCL appears as a round ovarian mass with a mean diameter of 3.0-3.5 cm, with well-defined, regular and thin walls (Figure 1)^(29)^.

Primarily, the clot forms like a fine fibrin network in the central cavity; subsequently, the coagulum in the cavity forms an organized reticular pattern^(29,30)^. After the luteo-lysis, the CL becomes “corpus albicans,” which is not always visible with ultrasound.

The fibrin in the central clot of the CL appears like solid septa and interdigitations forming a “complex mass”, entering into the differential diagnosis from ovarian neoplasm^(29,30,31,32)^.

For this reason, Yoffe et al.^(33)^ called HCL “the great imitator”, although there is always one posterior acoustic enhancement that allows to distinguish it from a solid lesion^(30)^.

**Figure 2:**
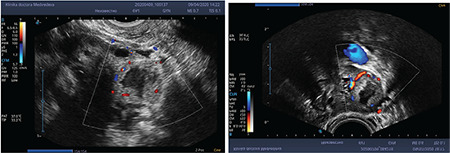
Doppler features of CLC (ring of fire)

Ding et al.^(34)^ observed 104 cases of hemorrhagic ovarian cyst, describing four different US patterns: 20.2% showed a diffused dense echo pattern, 24.0% showed a mixed pattern, 28.8% showed a sponge-like pattern and 27.0% showed a cystic pattern. They also observed a ring blood flow (ring of fire) with high velocity and low resistance without internal blood flow in 40% of cases (Figure 2).

Several studies have evaluated the sonographic and Doppler characteristics of the CL^(35,36,37,38)^ and the blood flow characteristics have been well defined.

Tamura et al.^(39)^ investigated the changes in CL blood flow during the luteal phase and early pregnancy. The relatively high resistance index (RI) during the late follicular phase declined with progression towards the luteal phase. By the mid-luteal phase, the RI was low, indicating a high blood flow to the CL. There was an increase in RI and therefore a reduction in the blood flow on regression of the CL. In women with luteal phase defects, the RI was significantly higher, indicating a decrease in blood flow. During pregnancy, the RI remains at the low mid-luteal phase level for the first 7-8 weeks and then increases once the CL regresses.

An abdominal or transvaginal US can also show hemorrhagic effusion into the abdominal cavity, especially at the lowest points such as the pouch of Douglas, the vesico-uterine pouch, and the iliac fossae. Hemorrhagic effusion can also be observed in the Morrison pouch^(6)^ and the paracolic lodges. The amount of effusion can vary from minimal to massive bleeding.

Examination of endometrium reveals a secretory pattern during the luteal phase and indicates an active production of progesterone.

Although ultrasonography is superior to computed tomography (CT) for making the diagnosis, the appearance of hemorrhagic cysts on CT scans depends on the age of the clot: blood from an acute hemorrhage has a high attenuation value, whereas blood from a previous hemorrhage has an attenuation value approaching that of water. In an acute setting, CT typically demonstrates a cystic adnexal mass with areas of high attenuation in the intramural and intracystic sites. Hemoperitoneum may also be present, with high-attenuation clot and blood accumulating in the pelvis. The pretreatment CT scan for ruptured corpus luteal cysts can suggest the necessity of surgical treatment based on image findings^(40,41)^.

### Differential Diagnosis

There are several pathologies that need a differential diagnosis with HCL. The pelvic pain is typical of many gynecologic disorders, such as ectopic pregnancy, PID, ovarian torsion, and several non-gynecologic diseases such as appendicitis, gastroenteritis, cystitis, and other urinary tract disorders^(42,43,44)^. Recently, a case of ruptured hemorrhagic ovarian cyst presenting an incarcerated inguinal hernia in an adult female was reported^(45)^.

The US differential diagnosis between some types of adnexal masses and complex malignant lesions is necessary^(29)^. Bleeding may be generally due to the rupture of an ectopic pregnancy, an infiltrating neoplastic disease, vascular diseases and traumas.

Clinical history and anamnesis of patients are very important for a differential diagnosis. In the event of a positive pregnancy test, the physician must investigate other nonspecific clinical findings of an ectopic pregnancy (e.g. lower abdominal pain, cervical motion tenderness, a palpable adnexal mass and uterine spotting). The earliest appearance of symptoms generally occurs in the sixth week after the last period. Peritoneal signs are indicative of intraperitoneal blood collection. Usually, the pain is alternating and spasmodic, followed by intervals free of symptoms^(46)^.

In most extrauterine pregnancies beta-hCG production is lower, the endometrium response is not stable, and spotting is common^(46,47,48)^. In 70% of ectopic pregnancies, the beta-hCG levels rise more slowly, reaching a plateau and even showing a decrease in serum levels. An abnormal beta-hCG pattern is highly suggestive of an ectopic gestation or a no longer intact gestation. Besides the unconventional rise in beta-hCG levels compared with normal pregnancies, ectopic pregnancy can be differentiated from a spontaneous abortion by a slower decrease in serum titer^(49,50,51,52)^. In a normally developing pregnancy, beta-hCG levels double every 1.5 days in the first 5 weeks of a regular gestation.

With a serum beta-hCG level of 1,500 mIU/mL, an intrauterine chorionic sac can be detected by transvaginal scan (TVS) presenting a double echogenic ring around a hypoechoic gestational sac called “comet sign”. Conversely, in patients with beta-hCG of 1,500 mIU/mL and more, an empty uterine cavity visualized by TVS with specifications as above can be taken as indirect proof of ectopic pregnancy.

The “tubal Ring” is a classic sonographic sign of ectopic pregnancy, represented by a hyperechogenic thick wall with anechoic content; only rarely it is possible to detect the yolk sac, which is often confused for a normal CL cyst^(53,54)^. Another significant sign of ectopic pregnancy is the “ring of fire”. Observed using the color Doppler, which is caused by vascularization of the placental blood flow of ectopic pregnancy. This sign, however, is similar to the one observed in the CL for the vascularization of the theca cells^(55)^. Therefore, these signs are not clear enough to differentiate an ectopic pregnancy from a CL. Frates et al.^(54)^ observed that the hyperechoic walls of the “tubal ring” of ectopic pregnancy reflected the hyperechoic area observed in intrauterine pregnancies at the initial stage, formed by the fusion of the trophoblast and the decidua. In several patients with ectopic pregnancy, the “tubal ring” has higher echogenicity than ovarian parenchyma and endometrium, and the walls of the CL show the same echogenicity, often appearing isoechoic or more often hypoechoic.

Visualization of the characteristic CL blood flow may aid in the diagnosis of ectopic pregnancy because about 85% of all ectopic pregnancies are found on the same side as the CL. This explains why in the majority of cases with proven ectopic pregnancy, luteal flow is detected ipsilaterally of the ectopic pregnancy. Luteal color or power Doppler flow may be used as a guide while searching for an ectopic pregnancy and could be called the “lighthouse effect” of CL, which directs the investigator to the color Doppler signals of the ectopic pregnancy^(53,54,55,56,57,58)^.

Vidakovic et al.^(59)^ reported a case of a ruptured CL cyst in early pregnancy with the authors suspecting an ectopic pregnancy, but after surgery, which confirmed HCL, a normal intrauterine pregnancy was found.

Torsion of the ovarian pedicle is also one of the most common complications of ovarian neoformations.

It usually occurs suddenly and can also affect normal adnexa (frequently the right adnexa). Venous stasis caused by torsion of the pedicle, like in the case of pelvic varices with the increase of vein blood pressure, can result in ovarian edema associated with HCL, as shown by Beyth et al.^(60)^.

The prevalent symptom is abdominal pain as in the HCL and the abdomen appears slightly treatable and tense. Color Doppler US is diagnostic if it shows the reduction or absence of blood flow.

Cysts presenting rapid growth may break due to abnormal vascularity in some parts of the wall.

If the cyst contains blood, differential diagnosis with rupture of HCL becomes particularly difficult. Endometriomas may increase in size in every menstrual cycle and rarely break with acute abdomen and spreading content, with the dissemination of endometrial tissue into the peritoneal cavity. US is the first-line imaging technique for diagnosis. Endometriomas can have a variety of US appearances. The “classic” endometriosis cyst has been described as a homogeneous, hypoechoic focal lesion within the ovary; the majority of the endometriomas show diffuse low-level internal echoes, multilocularity and hyperechoic foci in the wall^(61,62)^.

The differential diagnosis between HCL and endometriosis cysts, as well as the US appearance, is based on the US revaluation after the menstrual event, or at 4-6 weeks^(42)^.

The disappearance or regression of a previously observed cyst is indicative of CL, while the persistence of the cyst is indicative of endometriosis cysts.

In cases of PID, patients show inflammation signs such as fever, severe pain, and increased WBCs and other inflammatory markers; these signs are found only rarely in case of HCL.

In cases of PID, US examination of tubo-ovarian abscesses will usually show a complex adnexal structure, with increased vascular flow compared with endometriosis cysts, with thick walls and internal pus-like echoes with cellular debris. During transvaginal examinations, patients may exhibit tenderness over the area of the fluid collection^(61)^.

Classically, appendicitis diagnosis is based on symptoms, findings in a physical examination such as positive Blumberg sign, symptoms of acute abdomen, and severe leukocytosis. Acute abdomen and leukocytosis may both occur in case of HCL, but more often it is just a mild reaction without peritoneal muscle contracture and a modest increase in leukocytes levels^(24)^. US often shows an increase of the wall thickness and appendix edema, both typical of an inflammatory state.

If diagnostic doubt persists and the clinical symptoms appear in evolution, further imaging exams (e.g. CT and MRI) before performing a diagnostic laparoscopy are indicated.

### Corpus Luteum of Pregnancy

The CL of pregnancy is simply the persistence of a CL that accompanies conception. It is most prominent early in pregnancy and is critical to sustaining gestation in the first 8 weeks when the maximum progesterone secretion occurs. As pregnancy evolves, the CL gradually regresses and plays a negligible role in the final two trimesters.

With routine ultrasonographic examination during the first trimester, the discovery of an ovarian cyst has become relatively common at the beginning of pregnancy.

Most unilocular and anechoic ovarian cysts with thin borders during the first trimester are CL cysts. They are not generally present after the end of the first trimester. Except in case of complications, surgery should be avoided. The complications of these cysts are represented mainly by torsion, intracystic bleeding, and rupture. Adnexal torsion is frequently associated with ovarian stimulation for *in vitro *fertilization or with ovarian masses, mainly of functional origin. Operative laparoscopy has become increasingly common in pregnant women over the past decade.

Emergency surgery during the first trimester for complications of an ovarian cyst, especially before the ninth week of amenorrhea, is associated with a high rate of abortion. In the second part of pregnancy, fetal morbidity with prematurity caused by emergency surgery is considerable. The ideal period for scheduled surgery is probably the beginning of the second trimester when the abortion rate is minimized^(63)^.

A primary ovarian pregnancy is usually confused with tubal pregnancy, ruptured CL cyst, HCL, and ruptured endometrioma. This case presents the clinical and the histologic findings of a ruptured ovarian pregnancy, along with a ruptured CL cyst in the contralateral ovary.

Advances in laparoscopic technology and surgical techniques have overcome the technical difficulty of an enlarged gravid uterus^(64)^.

It certainly seems that progesterone may still play an important role postoperatively in the first trimester if surgery involves the adnexa and/or CL. However, by the seventh week, the placenta takes over the role of producing progesterone to support the pregnancy.

Ruptured CL cyst of pregnancy with massive hemoperitoneum is a rare but life-threatening disorder that can occur suddenly. Ovarian conservative treatment should be laparoscopic if the patient’s condition permits it.

### Therapy

Once the diagnosis of hemorrhagic CL with hemoperitoneum has been confirmed, it is necessary to establish a therapy. The decision about the treatment of CL includes the “wait-and-see” option, with a continuous follow-up of clinical, laboratory parameters, and US detection.

In most cases, keeping patients under observation and waiting for the remission of symptoms is enough. In rare cases, surgery may be required to stop bleeding because hemodynamic instability and deterioration of clinical status can occur. Surgical management should be laparoscopic.

An article published in 1984, in which 173 surgical cases were analyzed, it was concluded that in most cases a correct pre-surgical diagnosis allowed the avoidance of surgery^(1)^. Ho et al.^(6)^ showed that the use of surgery was significantly higher (100%) in the 1980s compared with cases examined between January 2001 and December 2003 (81.3%); however, the clinical manifestations of HCL were similar with those observed in the 1980s.

### Wait-and-see Attitude

With the improvement of diagnostic tools over time, physicians have increasingly opted for a wait-and-see approach. Most cases of ruptured CL cysts with moderate hemoperitoneum can be treated conservatively^(1,65)^.

During the observation period, it is important to continuously monitor US pelvic changes, hematocrit, and the symptoms reported by the patient.

The acute pain often subsides within the first 24 hours, and failure to improve could be a sign of worsening. Thus, during the observation period, it is recommended to perform another US evaluation and repeat the patient’s blood count, especially with signs of anemia (fainting, tachycardia, pallor, dyspnea, asthenia).

If Hgb values are stable or are maintained above 12 mg/dL, and if a US evaluation is compatible with the previous one, surgical treatment is not indicated.

If the symptoms disappear and US scans and Hgb values are unchanged, the patient can be discharged the same day with the recommendation of returning to the hospital immediately if pelvic pain or signs of anemia appear, or with the indication to be periodically followed by a general practitioner.

Pelvic US is also recommended after complete resolution of the clinical symptoms, preferably at the end of the following menstrual period.

### Drug Therapy

During the observation periods, physicians may prescribe drug therapy and supportive care; this, however, does not improve the outcome of the disease.

### Antifibrinolytic

Tranexamic acid is one of the most widely used drugs in this category. Tranexamic acid is a synthetic derivative of the amino acid lysine, which exerts its antifibrinolytic effect through the reversible blockade of lysine binding sites on plasminogen molecules. There are no clear indications for anti-fibrinolytic treatment, but we know from a few trials that systemic tranexamic acid administered at the outset for surgery reduces intraoperative blood loss, and if it is administered within 3 hours of any injury, it significantly decreases blood loss^(64)^.

### Analgesics

They are used on patients’ request to relieve painful symptoms, but pain does not always recede after the administration of these drugs.

### Supportive therapies

Liquid infusion is certainly a useful remedy in patients with HCL. They can be administrated to increase the circulating volume mass and prevent a drop blood pressure. Glucose-infusions can also be used because patients should be kept fasting in case of surgical treatment requirement.

### Transfusions

Sometimes blood transfusion is necessary, especially when Hgb and Hct are significantly reduced.

In cases of patients with bleeding disorders undergoing anticoagulant therapy, administration of fresh frozen plasma and vitamin K may be useful to replenish the missing coagulation factors^(23)^.

### Antibiotic Prophylaxis

Antibiotic prophylaxis is given to prevent bacterial superinfection of the pelvic effusion, which could result in real septic peritonitis. Antibiotic prophylaxis is conducted with broad-spectrum antibiotics active against the most common germs responsible for peritonitis. Prescription of antibiotics is made on an individual basis because the literature is lacking any evidence of their use in case of infection absence.

### Surgical Therapy

Surgical treatment was, in the middle of the last century, the first choice and consisted mainly of a laparotomy with oophorectomy. With the advent of laparoscopy, a minimally invasive approach is preferred to laparotomy, which however remains the first-choice method in the event of cardiovascular collapse^(66,67)^.

### Laparoscopy and Laparotomy

**Figure 3:**
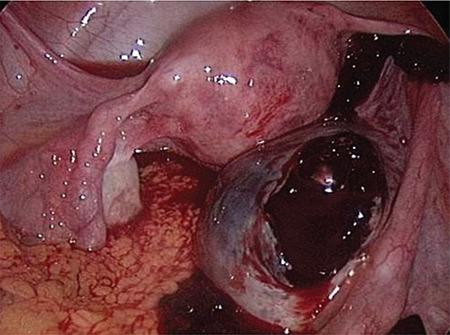
Laparoscopic view of ruptured CLC

Laparoscopy is the preferred surgical approach because it results in less morbidity than laparotomy (Figure 3). It is well known that the laparoscopy has several advantages compared with laparotomy, which has been replaced almost completely in gynecology during the last years^(65)^.

The first and most important advantage of laparoscopy is the type of incision, which is minimally invasive compared with laparotomic transverse incisions.

The hospitalization of the patient is reduced with laparoscopy compared with laparotomy (55±8 vs 98±14 hours) and post-operative pain is significantly reduced^(68)^.

Complications risks are lower in laparoscopy, which however is more operator dependent.

In the event of massive hemoperitoneum, autologous blood transfusions from blood recovered from the peritoneal cavity should also be considered, even with the laparoscopic technique^(69)^.

A limitation of the laparoscopic technique is the size of the cyst: if it exceeds the diameter size of 6-7 cm the indication to laparoscopy is limited to the surgeons’ experience (this event is however quite rare in cases of HCL).

During laparoscopy, three kinds of surgical options can be used:

• Cystectomy or enucleation of ovarian cysts (luteumectomy): The technique is preferable because it allows the preservation of ovarian function.

• Ovarian wedge-shaped excision.

• Oophorectomy or ovariectomy: this was the preferred technique in the past and resulted in the total loss of the ovary, often accompanied by loss of the ipsilateral fallopian tube.

### Women with Congenital or Acquired Bleeding Disorders

Women receiving anticoagulant therapy or with congenital disorders of the coagulation system have a higher risk of ruptured CL cysts. Surgical treatment is the traditional approach in this category of patients. There is still not enough evidence to support a surgical or expectant approach. According to some published case series, conservative treatment is the dominant trend in carefully selected patients with coagulopathies^(70)^.

In summary, the observational approach in hemodynamically stable patients could be the first choice option in most cases. There are no data comparing these two strategies in this patient population, but if there are no concerns about ongoing brisk bleeding or infection or malignancy, the risks of surgery are not warranted. In cases of continuing bleeding or the decision of a patient to choose active management, a laparoscopic approach should be suggested with ovary-sparing surgery, using minimal energy.

### Outcomes

There are scant data regarding the outcomes of ruptured ovarian cysts. Available studies include:

• In series of women with a ruptured ovarian cyst and hematoperitoneum, 15 of 78 were managed surgically^(65)^. Patients who underwent surgery had a more rapid Hgb decrease over 4 hours (1.7 vs 1.3 g/dL) and had higher transfusion rates than those managed conservatively (53 vs 11%).

• In another series of women with RCL, 58 of 70 women were managed with surgery and the remainder was managed with observation the study did not give rates of surgical complications, transfusions or recurrence^(66)^.

### Prevention of Recurrence

There are no known methods to prevent rupture of an existing ovarian cyst, except for surgical drainage or removal of the cyst. Patients with bleeding disorders or undergoing anticoagulant therapy have a higher risk of recurrence. Regular follow-up can minimize the risks. These patients often undergo surgery, with a higher risk of decreased ovarian function and adhesion formation, which consequently contributes to reduced fertility rates.

Thus, the possibility of preserving a future pregnancy is essential and patients need drugs such as estro-progestinics or GnRH analogues to prevent ovulation. Numerous studies have investigated the effects of OCs on follicular cyst development and ovulation. In general, current OCs resulted in the development of fewer follicular and correspondingly lutein cysts^(23)^.

## Conclusion

This review focuses on the pathophysiology, clinical presentation, diagnosis, and treatment options of HCL, also in patients with bleeding disorders or pregnancy complicated by CL cysts.

Rupture of CL is a common occurrence in women of reproductive age. Management is based upon patient characteristics, including the severity of symptoms, whether there is hemodynamic instability^(1)^. Currently, US is considered the gold standard technique in conjunction with clinical and laboratory findings for the diagnosis of HCL. HCL is clinically known to simulate several medical, surgical, and gynecologic conditions that cause acute abdomen^(42)^. The most important is ectopic pregnancy.

The “tubal ring” is a classic sonographic sign of ectopic pregnancy^(53,54)^.

Beta-hCG is essential for identifying pregnant patients and making a differential diagnosis with extrauterine pregnancy and intrauterine gestation or CL.

Observation is an adequate option in hemodynamically stable patients, without severe abdominal pain and in the presence of a small amount of pelvic fluid demonstrated by US. When a large amount of fluid is observed and/or in the presence of severe abdominal pain laparoscopy should be performed on admission. Direct laparotomy is mandatory in cases of circulatory collapse.

The decision on the treatment of CL includes the “wait-and-see” option with a continuous follow-up of clinical, laboratory parameters, and US detection.

During observation periods, drug therapy and supportive care are suggested. A careful pre-surgical diagnosis can often avoid the need for surgery.

Surgery may be rarely required to stop bleeding because hemodynamic instability and deterioration of clinical status can occur.

Patients with bleeding disorders or undergoing anticoagulant therapy have a higher risk of recurrence and often undergo surgery, with a higher risk of decreased ovarian function and fertility rates^(23)^.

This article provides an overview of HCL, with a focus on helping physicians to identify the clinical signs and sonographic features early, to quickly diagnose this condition, to choose the appropriate treatment for their patient, and to prevent recurrent episodes.
